# Can Working Conditions and Employees’ Mental Health Be Improved via Job Stress Interventions Designed and Implemented by Line Managers and Human Resources on an Operational Level?

**DOI:** 10.3390/ijerph18041916

**Published:** 2021-02-16

**Authors:** Magnus Akerstrom, Linda Corin, Jonathan Severin, Ingibjörg H. Jonsdottir, Lisa Björk

**Affiliations:** 1 Region Västra Götaland, The Institute of Stress Medicine, 413 19 Gothenburg, Sweden; linda.corin@vgregion.se (L.C.); jonathan.severin@vgregion.se (J.S.); ingibjorg.jonsdottir@vgregion.se (I.H.J.); lisa.m.bjork@vgregion.se (L.B.); 2 Occupational Medicine, School of Public Health and Community Medicine, Institute of Medicine, Sahlgrenska Academy at University of Gothenburg, 405 30 Gothenburg, Sweden; 3 Department of Sociology and Work Science, University of Gothenburg, 405 30 Gothenburg, Sweden; 4 Social Medicine, School of Public Health and Community Medicine, Institute of Medicine, The Sahlgrenska Academy at University of Gothenburg, 405 30 Gothenburg, Sweden

**Keywords:** sickness absence, workplace intervention, organisation, work environment, public sector, operational level, manager

## Abstract

Organisational-level interventions are recommended for decreasing sickness absence, but knowledge of the optimal design and implementation of such interventions is scarce. We collected data on working conditions, motivation, health, employee turnover, and sickness absence among participants in a large-scale organisational-level intervention comprising measures designed and implemented by line managers and their human resources partners (i.e., operational-level). Information regarding the process, including the implementation of measures, was retrieved from a separate process evaluation, and the intervention effects were investigated using mixed-effects models. Data from reference groups were used to separate the intervention effect from the effects of other concurrent changes at the workplace. Overall, working conditions and motivation improved during the study for both the intervention and reference groups, but an intervention effect was only seen for two of 13 evaluated survey items: clearness of objectives (*p* = 0.02) and motivation (*p* = 0.06). No changes were seen in employees’ perceived health, and there were no overall intervention effects on employee turnover or sickness absence. When using operational-level workplace interventions to improve working conditions and employees’ health, efforts must be made to achieve a high measure-to-challenge correspondence; that is, the implemented measures must be a good match to the problems that they are intended to address.

## 1. Introduction

Sickness absence is a major concern within the public sector, both in Sweden and in other countries. Many workplaces, especially those within the healthcare sector, experience high rates of mental health problems such as burnout, anxiety, and depression, and the work environment has been shown to be an important contributing factor to this [1,2,3,4,5]. High sickness rates among healthcare employees worsen the current and future labour shortage within the healthcare sector [6,7]. Multiple factors in the workplace have been recognised as determinants for employees’ stress-related mental health problems, including burnout, such as high job demands; low job control; low social support; effort–reward imbalance; low organisational, procedural, or relational justice; organisational change; job insecurity; temporary employment status; atypical working hours; poor psychosocial safety climate; bullying; and role stress [8,9,10,11]. Although numerous studies have described the prevalence and determinants of burnout and other mental health problems due to poor working conditions, there is still only limited knowledge of effective methods for improving these adverse conditions, and consequently the employees’ mental health, especially on an organisational level [12,13].

Organisational-level workplace interventions have been recommended on a theoretical basis to prevent stress-related mental health problems among employees, because such interventions address “the cause of the causes” of work-related stress within the organisation rather than strengthening the individuals populating them [13,14,15,16,17], and thus probably provide more long-term effects [18]. However, evaluations of organisational-level interventions have shown inconclusive results [19,20,21,22]. To understand these inconsistencies, qualitative process data have been used to better understand the importance of the context [13,17,23,24]. Another complementary way to disentangle these inconsistencies is to ensure that the effect evaluation includes not only organisational outcomes such as sickness absence and employee turnover, but also intermediate effects such as predictors of employees’ health and stress reactions [25,26,27] as well as multilevel predictors of the working conditions (i.e., both on an organisational and a work task level). Thus, including a wider range of outcome measures, representing not only organisational outcomes but also effects on both the “causes” and the “cause of causes” of the organisational outcomes evaluated [13,14,16,17], might be another way to increase the possibility of higher consistency in the evaluation of organisational-level interventions, and consequently to increase the knowledge of plausible effects of these interventions.

One way to evaluate the effects of organisational-level interventions is to measure several aspects of plausible changes that could contribute to explaining mechanistic pathways between adverse working conditions and sickness absence. In this context, the widely-known job demands–resources (JD-R) model could preferably be used to measure work conditions [28]. The JD-R model has previously been used to investigate the impact of the work environment on different outcomes such as burnout [29,30,31], commitment [32,33], engagement [31,34], task enjoyment [32], absenteeism [28], and employee turnover [35]. Using the JD-R model as a framework for organisational-level intervention evaluations makes it possible to evaluate the total effect of an intervention by including not just the organisational outcomes, such as employee turnover and sickness absence, but also effects on the “causes” (i.e., working conditions) and predictors of the employees’ health and motivation, which represent intermediate factors in the process between adverse working conditions and organisational outcomes.

Organisational-level job stress interventions are designed and implemented in a variety of ways. Most of the interventions evaluated in the literature were initiated, designed, and implemented by researchers or experts, and a participatory approach has often been used to achieve a high fit to the context [36,37,38]. On the other hand, job stress interventions are also initiated, designed, and implemented by the organisations themselves, without support from researchers or experts, often with the aim of preventing negative organisational outcomes from poor working conditions and mental health among the employees [13].

In 2017 and 2018, the management of a large Swedish administrative region within the public sector (approximately 55,000 employees, with approximately 85% working in the healthcare sector), initiated an organisational-level intervention aimed at reducing sickness absence. As a part of this job stress intervention, line managers together with their respective human resources (HR) partners were encouraged to assess the need for improvement within their work environment. Those who saw such a need were invited to apply for funds to implement measures, preferably on an organisational level, aimed at addressing these needs. By investigating the effectiveness of different approaches to interventions, including those designed and implemented by the employers themselves, improvements in the design and implementation processes of organisational-level interventions could be achieved.

### Aim

This study aimed to evaluate the effectiveness of an organisational-level job stress intervention, performed by managers and HR on an operational level, by investigating the intervention effects on working conditions, motivation, health, employee turnover, and sickness absence.

## 2. Materials and Methods

### 2.1. Setting and Intervention Design

**Figure 1:**
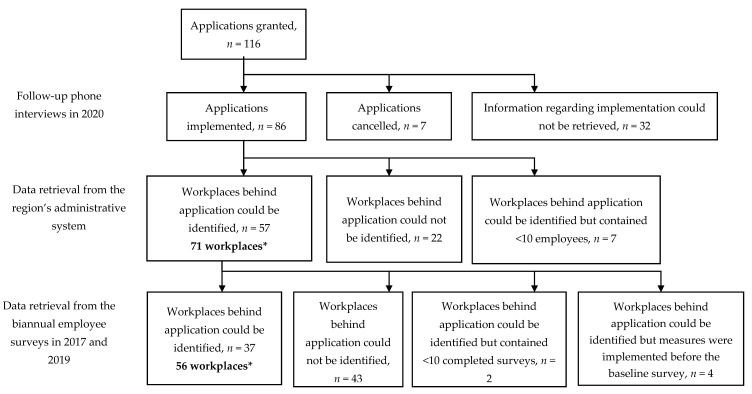
Flowchart describing the number of granted and implemented applications in the intervention together with the number of workplaces (i.e., intervention groups) identified and eligible for the evaluation of intervention effects. * Applications could contain more than one measure; therefore, applications (and measures) could involve more than one workplace and workplaces could be part of more than one application.

The setting and intervention design are described in detail elsewhere [39]. Briefly, this operational-level intervention was carried out in one of the 21 administrative regions responsible for healthcare, culture, and transportation in the public sector of Sweden. As a part of the intervention, line managers together with their respective HR partners were encouraged to assess the need for improvement within their work environment and apply for funds to implement measures aimed at addressing these needs. According to the criteria used to distribute the funds, the measures should be proactive and affect the employees’ work environment by targeting how work was organised and/or executed, rather than by strengthening individual employees [13,14,16,17,40,41,42]. As support, each line manager had their respective HR partner and the possibility to consult the internal occupational health services free of charge. In total, 154 applications containing 209 suggested measures were submitted. Common types of measure suggested were lectures and workshops, often aiming to inspire, motivate, and/or support individual employees in improving their lifestyle or providing them with personal strategies to manage their situation or teambuilding; for example, to improve the group dynamics or to strengthen the cooperation between employees at the workplace. Other common measures included work environmental analyses (e.g., assessments to identify challenges or developing action plans on how to improve the work environment), manager support, and structural changes including schedule improvements or improvements involving day-to-day routines within the operation [39]. Most workplaces (69%) were healthcare workplaces; 18% were administrative units, 8% were service and maintenance units, and 5% were management workplaces [39]. The number of applications granted and implemented, together with the number of workplaces included in this effect evaluation, are given in Figure 1. The measures were implemented between early in 2017 and late in 2018.

To separate plausible effects of the intervention from the effects due to other concurrent changes in the workplaces [43,44], data were also collected for each corresponding operational area and department (i.e., the next two higher levels in the organisational hierarchy, which roughly comprised the management board, departments, operational areas, and workplaces) during the time of the intervention. Reference data on working conditions, motivation, and health were available on the same organisational level as the intervention groups, resulting in 247 reference groups (i.e., workplaces) nested within 18 operational areas and 10 departments. Employee turnover and sickness absence data were only available on an aggregated level; that is, the average for the respective operational areas and departments (with data for the intervention groups subtracted).

### 2.2. Effect Evaluation

#### 2.2.1. Survey Data on Working Conditions, Motivation, and Health

**Figure 2:**
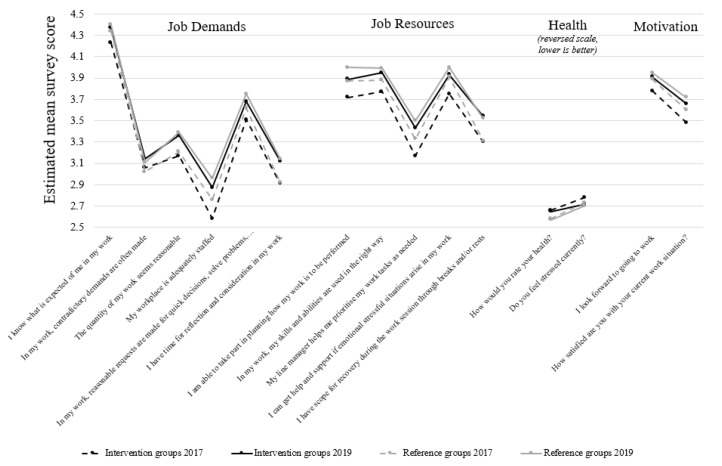
Estimated mean survey scores for the evaluated items representing job demands, job resources, health, and motivation, stratified by intervention status.

To evaluate the intervention effect on working conditions, motivation, and health, data were collected from the region’s employee survey. Baseline data were taken from the survey conducted in September 2017 (overall regional response rate: 73%), and follow-up data from the survey conducted in October 2019 (overall regional response rate: 77%). Aside from background information, the survey contained 53 items concerning conditions at the workplace and how the respondents perceived their work environment, health, and the occurrence of discrimination. Responses were made using a five-point Likert scale. Fifteen items were selected from the survey representing working conditions such as job demands (6 items covering quantitative demands, cognitive demands, role conflicts, and clearness in objectives) and job resources (5 items covering control, competence, support, and possibility for recovery), as well as motivation (2 items) and health (2 items) (Figure 1 and Figure 2). The evaluation used completed surveys from the employees within each intervention group; i.e., employees at workplaces that had implemented one or more measures (a total of 3200 surveys from 2017 and 2019 combined).

#### 2.2.2. Register Data on Employee Turnover and Sickness Absence

Monthly data on total employee turnover, sickness absence, and short-term sickness absence (≤14 days) between January 2016 and March 2020 were retrieved from the region’s administrative employee system for the intervention groups and their respective reference groups. Employee turnover was expressed as the percentage turnover (number of individuals leaving the workplace divided by the total number of employees in each group and month), and sickness absence was calculated as the percentage absence on a group level based on the number of hours of absence due to sickness divided by the total number of hours the group was expected to work each month (vacation, parental leave, and caring for sick children deducted).

### 2.3. Analytical Strategy

The Shapiro–Wilk test and visual inspection of histograms were used to test the effect measures for normality. Parametric methods on untransformed data were used in the subsequent analyses, because the assumption of normality was judged to be plausible. Statistical significance was set at *p* < 0.05, and two-sided confidence intervals were used.

The intervention effects were evaluated in three steps: (i) analyses of the overall intervention effects; (ii) analyses of differences in the intervention effects between subgroups and changes over time; and (iii) when applicable, analyses of workplace-specific intervention effects.

In the first step, overall effects were estimated for the intervention groups using a random-intercept or random coefficient model (Proc Mixed in SAS version 9.4; SAS Institute, Cary, NC, USA) with group as the random effect. The models used to evaluate the different effect measures are specified below. Hypothesis testing for fixed effects was performed using Wald tests, and tests of random effects were performed using likelihood ratio tests.

When analysing overall intervention effects on working conditions, motivation, and health, concurrent effects for the reference groups could be analysed simultaneously in the model by adding information on operational areas and departments as random effects. Additionally, fixed effects for the year (2017 or 2019), intervention status (dummy variable; 1 for the intervention groups and 0 for the reference groups), and the interaction between year and intervention status were added to analyse the overall effect of the intervention.

When analysing the overall intervention effect on employee turnover and sickness absence, time (nested within workplaces) was added as a random effect, and a first-order autoregressive correlation structure (AR) [1] was used to account for correlations between repeated measurements of the same workplace. Additionally, fixed effects for year (continuous) and month (categorical: 1–12) were added to the model to control for time trends and seasonality, and a dummy variable for the intervention (0 up to the beginning of the intervention and then 1) was added to analyse the overall effect of the intervention. Any concurrent effects for the reference groups (the respective operational areas and departments) were determined separately due to the structure of the collected data (i.e., data on a higher organisational level).

In the second step, differences in the intervention effects between subgroups and changes over time were investigated either by adding interaction terms between the intervention variable and variables for workplaces or background data concerning the implemented measures, such as level and perspective (organisational, group or individual and promotive, preventive or rehabilitation, respectively), or by stratifying the analyses according to the above. To investigate delayed intervention effects on employee turnover and sickness absence, an intervention effect with a time lag of 1, 3, or 6 months after the start of the intervention was added to the models.

In the third and final step, intervention effects on employee turnover and sickness absence for the individual workplaces and their respective reference groups were estimated using Box–Jenkins autoregressive integrated moving average (ARIMA) time series methodology [45,46] to discover whether the size and/or direction of the intervention effect differed between the different workplaces within the intervention. An ARIMA model including seasonal components was derived for each measure and workplace using the Time Series Modeler in version 25 of SPSS Statistics (IBM, Armonk, New York, NY, USA). The intervention variable was then added to these models to analyse the effect of the intervention. Corresponding workplace-specific analyses of the intervention effect on working conditions, motivation, and health could not be performed due to the low number of repeated measurements (data from only two surveys, rather than monthly data, as was available for employee turnover and sickness absence).

### 2.4. Ethics Approval and Consent to Participate

This study was approved by the regional ethics committee in Gothenburg (reference: 911–18), and the workplaces agreed to participate after giving their informed consent.

## 3. Results

**Figure 3:**
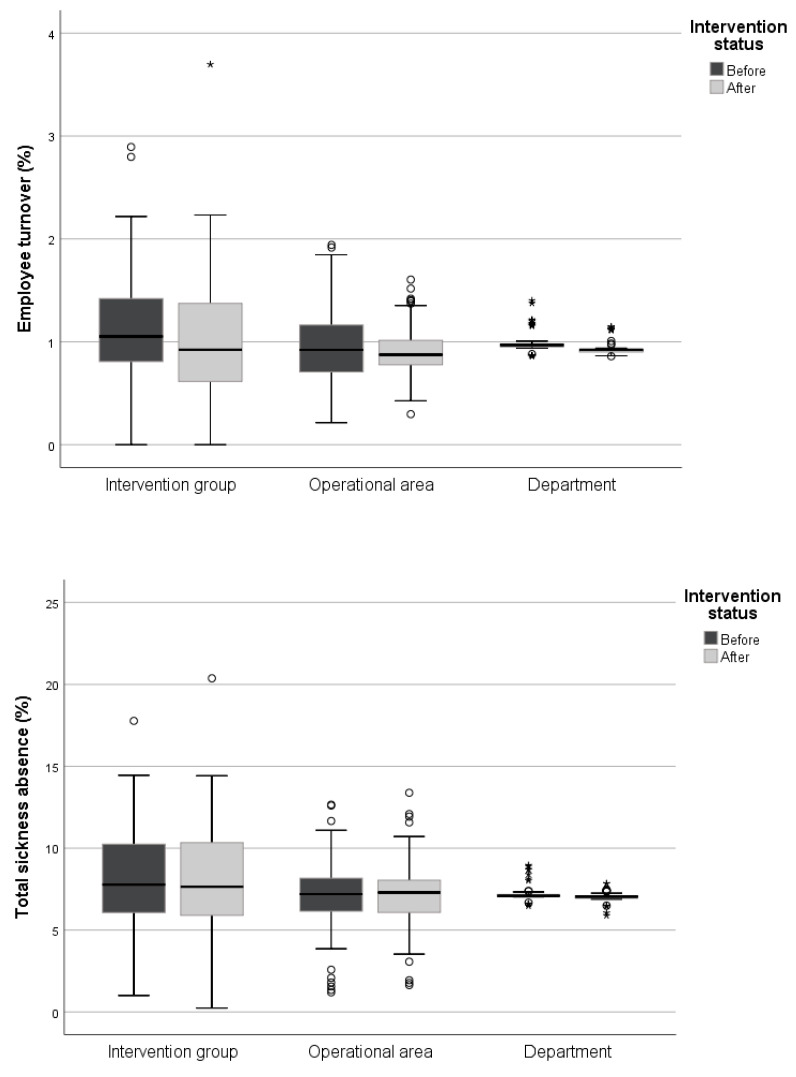

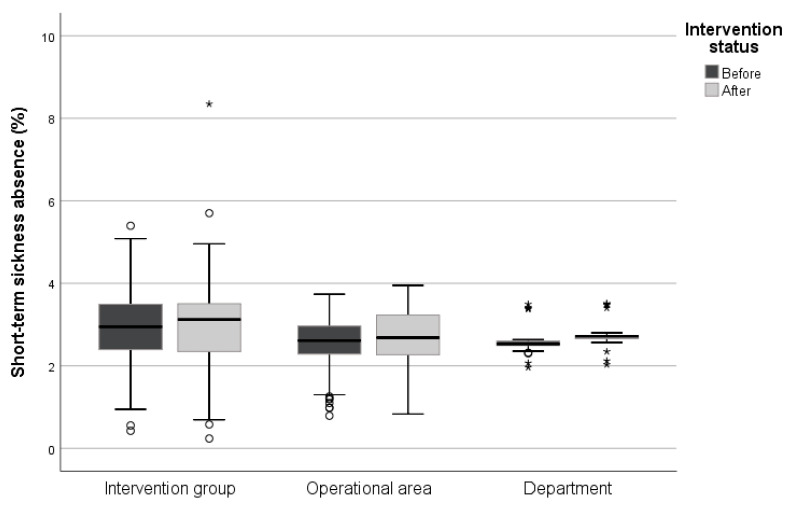
The distribution of the workplace means employee turnover and sickness absence before and after the intervention.

Average levels on working conditions, motivation, health, employee turnover (%), and sickness absence (total and short-term in %) for the intervention and reference groups, before and after the start of the intervention, are given in Figure 2 and Figure 3.

### 3.1. Overall Intervention Effects on Working Conditions, Motivation, and Health

No overall intervention effects were seen for 13 of the 15 evaluated items. However, a statistically significant positive overall intervention effect (*p* = 0.02) was seen for the survey item “I know what is expected of me in my work”, representing the job demand clearness in objectives. The estimated mean survey score increased from 4.23 to 4.40 (*p* < 0.001) among the intervention groups (when estimated in the models described above) and from 4.34 to 4.37 (*p* = 0.06) among the reference groups (i.e., the workplaces which did not implement measures within the same operational area). There was also a tendency for a positive intervention effect (*p* = 0.06) for the item “I look forward to going to work”, representing motivation, with an increase from 3.78 to 3.91 (*p* < 0.001) among the intervention groups and from 3.89 to 3.95 (*p* = 0.07) among the reference groups.

The result on the overall intervention effects for the 15 evaluated items did not change when information was included in the models regarding type of workplace, type of organisation, the level or perspective of the implemented measures, whether measures were performed on more than one level, and whether there was a measure-to-challenge correspondence; that is, whether the implemented measures were a good match to the problems they were intended to address [39].

### 3.2. Differences in Working Conditions, Motivation, and Health Between Intervention Groups and Reference Groups, and Their Development over Time

At the baseline survey, the intervention groups had significantly lower survey scores than the reference groups for three items regarding motivation (“I look forward to going to work”, 3.78 versus 3.89, *p* = 0.01) and support (“My line manager helps me prioritise my work tasks if needed”, 3.17 versus 3.33, *p* = 0.04, and “I can get help and support if emotional stressful situations arise in my work”, 3.75 versus 3.91, *p* = 0.02). Furthermore, there was a tendency for lower survey scores on another three items regarding health (“How would you rate your health?”, 2.66 versus 2.58 (reversed scale—lower is better), *p* = 0.07), motivation (“How satisfied are you with your current work situation?”, 3.48 versus 3.60, *p* = 0.05) and clearness in objectives (“I know what is expected of me in my work”, 4.23 versus 4.34, *p* = 0.08). At the follow-up survey in 2019, no statistically significant changes were seen between the intervention and reference groups, but there was a tendency for lower survey scores among the intervention groups than among the reference groups for one item regarding health (“How would you rate your health?”, 2.65 versus 2.57 (reversed scale—lower is better), *p* = 0.05).

Between the two surveys in 2017 and 2019, both the intervention groups and the reference groups improved on all items regarding their working conditions and motivation, (*p* = 0.01—*p* < 0.001), but no statistically significant changes were seen for the items regarding their perceived health (*p* = 0.1—*p* = 1.0).

### 3.3. Overall Intervention Effects on Employee Turnover and Sickness Absence

No statistically significant intervention effects could be seen for total employee turnover or sickness absence, despite an increased employee turnover for the departments and a decreased sickness absence for both the operational areas and the departments during the time of the intervention (Table 1). There was a statistically significant decrease of 0.2 percentage points in the short-term sickness absence for the intervention group, but a simultaneous decrease was also seen for both the operational areas (0.13 percentage points) and the departments (0.08 percentage points) (Table 1). Introducing a time lag of 1, 3, or 6 months to detect any potential delayed effects did not change the result. In addition, there were no interaction effects between the intervention variable and time for employee turnover or sickness absence.

There was a statistically significant interaction between the type of organisation and the intervention variable for short-term sickness absence (*p* = 0.003), but not for total sickness absence or employee turnover. When stratified by type of organisation, a positive intervention effect was seen for the intervention groups from primary health care (β = −1.4, 95% CI: −0.49—−2.2, *p* = 0.002, *n* = 4) and a tendency for a positive effect among intervention groups from the small hospitals (β = −0.46, 95% CI: −0.95–0.03, *p* = 0.07, *n* = 11), but no effect was seen for intervention groups at the large hospital (β = −0.09, 95% CI: −0.32—0.13, *p* = 0.4, *n* = 51) or at internal services (β = 0.15, 95% CI: −0.58—0.86, *p* = 0.7, *n* = 5). No differences in intervention effects could be seen between the intervention groups or the type of workplace.

In terms of the influence of workplace size, the intervention effect decreased with increasing numbers of employees (−0.03%/employee, *p* = 0.01) for total sickness absence.

Neither the level of described work environmental challenges (organisational or group) nor the level or the perspective of the implemented measures (organisational, group or individual and promotive, preventive and rehabilitation, respectively) affected the intervention effect for employee turnover or sickness absence. The presence or absence of a measure-to-challenge correspondence (i.e., whether the implemented measures were a good match to the problems they were intended to address) also did not affect the intervention effects. However, there was a significant influence on the intervention effect on sickness absence when measures were implemented on more than one level (*p* = 0.02 for total sickness absence, *p* = 0.07 for short-term sickness absence). Stratifying for those who had only implemented measures on one level (*n* = 57) and those who had implemented measures on more than one level (*n* = 14) resulted in intervention effects for total and short-term sickness absence (in %) of −0.37 (95% CI: −0.93—0.20, *p* = 0.2) and −0.17 (95% CI: −0.38—0.042, *p* = 0.1) for measures on one level and −0.81 (95% CI: −2.5—0.85, *p* = 0.3) and −0.34 (95% CI: −0.77—0.093, *p* = 0.1) for measures on more than one level, respectively.

### 3.4. Workplace-Specific Intervention Effects on Sickness Absence and Employee Turnover

**Figure 4:**
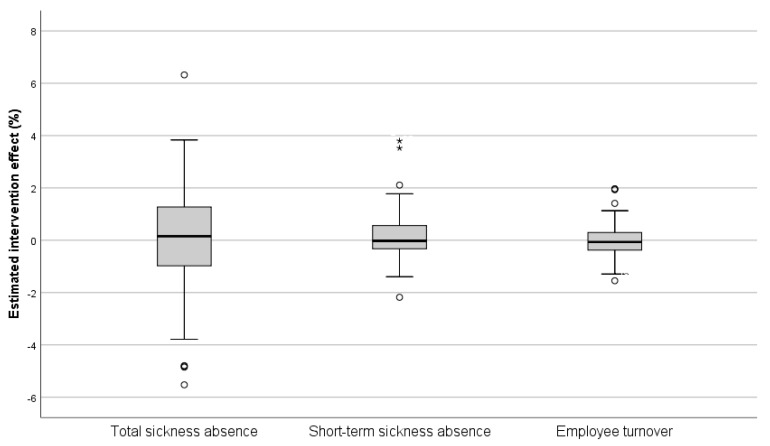
Distribution of estimated workplace-specific intervention effects on employee turnover and sickness absence among the 71 participating workplaces.

The intervention effects differed between the individual intervention groups (Figure 4), and simultaneous positive and negative concurrent changes were also seen for the operational areas and the departments. For total sickness absence, approximately 11% of the intervention groups had a statistically significant positive intervention effect, while approximately 15% had a significant negative effect, i.e., a negative development during the time of the intervention. When the result was adjusted for the concurrent development of the reference groups, the percentage with a negative effect decreased to 13% (Table 2). Corresponding results for short-term sickness absence and employee turnover were 6% and 4% for positive intervention effects and 8% and 3% for negative effects, respectively (Table 2).

## 4. Discussion

The present study reports on a job stress intervention that was initiated, designed, and implemented by line managers on an operational level. It contributes to the emerging issue of finding efficient approaches for improving the working conditions and decreasing sickness absence in real-life settings [12,36]. The analysis used sophisticated evaluation methods that both included the context and separated the intervention effect from concurrent changes. Taking the context into consideration and singling out intervention effects are important steps to overcome previously identified challenges in the evaluation of organisational-level interventions [17,38,47].

### 4.1. Overall Intervention Effects

This advanced evaluation approach only revealed positive intervention effects for single survey items within job demands (clearness in objectives) and motivation (“I look forward to going to work”). Consequently, no intervention effects could be seen for either job resources, health, employee turnover or sickness absence. Evaluations of organisational-level intervention have shown inconclusive results in the past, with some but not all evaluations reporting positive effects [19,20,21,22,38,48].

One possible explanation for the absence of clear intervention effects might be the low measure-to-challenge correspondence when designing measures, as demonstrated in the process evaluation [39]. Although the intervention reached the intended target group, only half of the applications for funds contained a measure-to-challenge correspondence, and only a third of the suggested measures were on an organisational level as had been intended [39]. The absence of intervention effects due to low adherence to the intention of the intervention has also been seen in the evaluation of other organisational-level interventions, and has been connected to both a lack of knowledge regarding these issues within the organisation and insufficient preconditions for managers to work efficiently with such issues [38,49].

The general positive development regarding work conditions and motivation for both intervention and reference groups might also have masked an intervention effect, because despite the use of sophisticated statistical methods, it is not possible to assess whether the intervention groups would have followed the general development if they had not been participating in this intervention.

By using the JD-R model as a framework for the evaluation, we were able to gain further insight into the mechanism behind a potential intervention effect on organisational outcomes such as employee turnover and sickness absence. In our case, no intervention effects were seen for the organisational outcomes investigated, but there was some (albeit inconsistent) evidence for intervention effects on single items within evaluated job demands and motivation which could be used to explain the relationship between working conditions and employees’ health and well-being [28]. These effects should not be overlooked, because earlier studies have stressed the importance of focusing on optimising job demands; merely improving job resources is not necessarily enough to reduce the risk of burnout or sickness absence [35,36,50]. In addition, because decreasing job demands is particularly difficult in the healthcare sector, which is characterised by unlimited needs and limited resources [51], even seemingly small decreases in job demands, such as in this intervention, can be considered to be of great value.

### 4.2. Factors Affecting the Intervention Effect

Overall, none of the factors included in this study were found to modify the intervention effects on working conditions, motivation, and health. However, our results have identified factors affecting the development of sickness absence among the intervention groups. Firstly, the context where the measures were implemented affected the outcome of the organisational outcomes, in terms of both the type of organisation and the size of the workplace. It is well-known that there are differences in working conditions between different sectors or types of organisation within the public sector [52] that might result in different possibilities to efficiently implement an intervention, but evidence for an impact of workplace size is less conclusive [53,54]. The nature of the implemented measures also influenced the intervention effect for sickness absence, in that workplaces implementing measures on more than one level had a more positive development; this has also been seen by others [13,20,42,55]. As argued before [16,18,56], this indicates that organisational-level measures should not completely replace individual-level measures in organisational-level interventions, but rather promote a multilevel approach to complex work environmental challenges within the workplace. As mentioned above, no effect was seen for the level (individual, group, or organisational) or perspective (promotive, preventive, or rehabilitation) of the implemented measures, as might have been expected [13,14,15,16,17]. However, stratifying by the level or perspective of the measure did not take the measure-to-challenge correspondence into account, which may have affected the result. Consequently, these differences in both the implemented measures and the workplaces in which the measures were implemented could be one reason for the large variation in intervention effects for the individual workplaces demonstrated in this study.

### 4.3. Methodological Considerations on Implementing and Evaluating Operational-Level Interventions

In the absence of thorough knowledge on optimal design and implementation of workplace interventions, different approaches are being applied that consider one or more of the aspects highlighted by research as being important success factors. Operational-level interventions where the line managers together with their respective HR partners have the authority to design and implement measures themselves are likely to lead to high management support [17,57,58,59], fit to the workplace context [17], and integration of the measures into existing structures [60,61]. Meanwhile, the approach requires knowledge of the psychosocial work environment to be able to tailor the measures for the specific group, work environment challenge, and context [62,63]. It also requires knowledge of change management if the measures are to be implemented in an efficient and successful way [64,65], which the line manager or the organisation as such may or may not possess. Our evaluation also clearly shows that there was a low measure-to-challenge correspondence when it came to designing organisational-level measures tailored to the specific group and context [39]. Thus, in order to increase the efficiency of operational-level interventions, line managers need support in designing and implementing measures that correspond both to the unique work environmental challenge at the workplace and to the context in which the measure should be implemented. Another important aspect is to allocate enough resources within the workplace to enable the work to be done. This could be achieved by implementing measures early in the process aimed at temporarily reducing the job demands and/or adding additional resources [66]. Furthermore, highly structured systematic occupational health and safety management, and beneficial public policies, also help managers to create organisational working conditions which enable the work to be done [52,60].

Randomised controlled trials are often considered to be the gold standard for evaluating the effectiveness of different treatments or approaches, but they are not sufficient in real-life settings [14]. The framework for evaluating an organisational-level intervention needs to be designed in a way that can take into consideration the context of the intervention groups, the implementation, and the effect of other concurrent changes at the workplace. The result of the process evaluation [39] and the large variation in intervention effects between the intervention groups further stress this conclusion. It is also of greatest importance to include a comparison with reference groups in the evaluation [19,67], in order to rule out other possible causes of change in the outcome. Moreover, a comparison with reference groups could allow the detection of additional intervention effects, because a non-effect may be seen as an intervention effect in comparison with the development of the effect measures in the reference groups, and vice versa. This was seen in the present study, when the percentage of workplaces with a negative development decreased after comparing with the results of the reference groups.

In this study, we attempted to consider different parts of the pathway between adverse working conditions and organisational outcomes, including turnover and sickness absence. However, because limited effects were generally seen following the intervention, the plausibility of capturing different effects related to the pathway—and thus, mechanisms of change—was low. Furthermore, most of the measures included were multifactorial and complex, including sickness absence, which could be considered as a passive and individual strategy for coping with work environmental issues. An intervention might affect this individual strategy in a different way, compared to survey results on perceived working conditions [67,68]. The relationship between specific working conditions and sickness absence is not fully understood. Although a relationship has been established in cross-sectional studies between specific working conditions and sickness absence, this relationship seems to be affected by the context of the workplace [69]. Thus, more research is needed to further explain the mechanism of change of organisational-level interventions.

### 4.4. Strengths and Limitations

A strength of this study is the use of survey and register data from the employer’s administrative systems on working conditions, health, motivation, employee turnover, and sickness absence. Another strength is the framework of this evaluation, with both a qualitative process evaluation and a quantitative effect evaluation including effects on both organisational outcomes and factors predicting intermediate effects such as employees’ working conditions, health, and motivation [17,55,56,62]. The evaluation was performed with sophisticated methods which enabled us to separate the intervention effect from the effects of other concurrent changes in the work environment [43,44].

Although register data from the employer are of higher quality than self-assessed data, especially for employee turnover and sickness absence, the use of these data in the study also brought some challenges. Data on working conditions were restricted to items used in the regions’ employee survey and potential effects not included among these items could not be investigated. We were not able to include all participating workplaces in the evaluation, because the administrative personnel system did not completely match the workplaces’ organisational structure, and consequently data could not be retrieved for all intervention groups. We were also limited to aggregated data on a workplace level, which did not enable us to take into account changes caused by employee turnover. However, the effect on employee turnover was also assessed, and no significant change was seen during the time of the study. Another limitation of this study was that we were constrained to using reference groups at a higher organisational level than the intervention groups for the analyses of sickness absence and employee turnover. It was impossible to find matched control groups or to retrieve information about reference groups at the same organisational level as the intervention groups due to technical limitations in the regions’ administrative employee system. If reference groups at the same organisational level had been available, comparisons between intervention and reference groups made within the same models could have been used (as in the analyses of the working conditions) instead of comparing the results of separate models. Finally, for the data on working conditions, health, and motivation among the employees, we were limited to surveys distributed in September 2017 and October 2019 to evaluate intervention effects from measures performed from early in 2017 to late in 2018. Thus, because the pre- and post-measurements were not adjusted to the individual measures (as in the evaluation of effects on employee turnover and sickness absence), and due to the possibility of there having been a lag in effects and/or that the intervention might have only produced short-term effects, the use of fixed pre- and post-measurements might have affected the possibility to detect an intervention effect. To reduce this risk, workplaces implementing measures before the pre-measurement were excluded.

## 5. Conclusions and Practical Implications

This study shows that interventions aimed at improving work conditions and decreasing sickness absence in complex organisations are highly contextual, and thus both the implementation and the measuring of plausible effects of these interventions are associated with great challenges. As well as providing financial resources, the employer also needs to ensure that the line managers responsible for implementing the measures are given adequate preconditions, knowledge, and/or access to support from expert functions. Combining the findings from the process evaluation with the absence of distinct intervention effects from the present study clearly shows that the line managers and their HR partners can analyse their work environment to identify the work environment issues. However, they need support in designing measures tailored to the specific work environment challenges at hand, as well as support in the change management process, to increase the effectiveness of operational-level job stress interventions.

## Figures and Tables

**Table 1:** Estimated intervention effects on sickness absence and employee turnover in the intervention groups, and concurrent changes in the reference groups.

Effect Measures	Intervention Group		Operational Area ^a^		Department ^a^	
	β (95% CI)	*p*-Value	β (95% CI)	*p*-Value	β (95% CI)	*p*-Value
Sickness absence (%)	−0.43 (−1.0–0.14)	0.14	−0.29 (−0.56–−0.015)	0.04	−0.17 (−0.26–−0.077)	<0.001
Sickness absence ≤14 days (%)	−0.20 (−0.39–−0.007)	0.04	−0.13 (−0.23–−0.023)	0.02	−0.084 (−0.12–−0.046)	<0.001
Employee turnover (%)	0.071 (−0.18–0.32)	0.58	0.050 (−0.079–0.18)	0.45	0.030 (0.012–0.049)	0.001

^a^ Intervention group excluded.

**Table 2:** Workplace-specific intervention effects on sickness absence and employee turnover expressed as the number of the 71 intervention groups with a statistically significant (*p* < 0.05) intervention effect. In total and after adjustment for the concurrent development within the reference groups (removing workplace-specific interventions effects that could be explained by the general development in the operational area or department instead of the intervention).

Intervention Effects	Total Sickness Absence		Short-Term Sickness Absence		Employee Turnover	
	Total	Adjusted for Reference Groups	Total	Adjusted for Reference Groups	Total	Adjusted for Reference Groups
Positive intervention effect (*n*)	8	8	4	4	3	3
Negative intervention effect (*n*)	11	9	6	6	3	2

## Data Availability

Data available on request.
